# Suicidality among adolescents engaging in nonsuicidal self-injury (NSSI) and firesetting: the role of psychosocial characteristics and reasons for living

**DOI:** 10.1186/s13034-015-0068-1

**Published:** 2015-09-28

**Authors:** Alicia K Tanner, Penelope Hasking, Graham Martin

**Affiliations:** School of Psychological Sciences, Monash University, Melbourne, Australia; School of Psychology and Speech Pathology, Curtin University, Perth, WA 6845 Australia; Department of Psychiatry, Monash University, Melbourne, Australia; Department of Psychiatry, The University of Queensland, Brisbane, Australia

**Keywords:** Suicidal ideation, Suicide, NSSI, Firesetting, Adolescence

## Abstract

**Background:**

Co-occurrence of problem behaviors, particularly across internalizing and externalizing spectra, increases the risk of suicidality (i.e., suicidal ideation and attempt) among youth.

**Methods:**

We examined differences in psychosocial risk factors across levels of suicidality in a sample of 77 school-based adolescents engaging in both nonsuicidal self-injury (NSSI) and repeated firesetting. Participants completed questionnaires assessing engagement in problem behaviors, mental health difficulties, negative life events, poor coping, impulsivity, and suicidality.

**Results:**

Adolescents endorsing suicidal ideation reported greater psychological distress, physical and sexual abuse, and less problem solving/goal pursuit than those with no history of suicidality; adolescents who had attempted suicide reported more severe NSSI, higher rates of victimization and exposure to suicide, relative to those with suicidal ideation but no history of attempt. Additional analyses suggested the importance of coping beliefs in protecting against suicidality.

**Conclusions:**

Clinical implications and suggestions for future research relating to suicide prevention are discussed.

## Background

Nonsuicidal self-injury (NSSI; the purposeful destruction of body tissue without conscious suicidal intent) has emerged as a prominent threat to psychological functioning in adolescence, with prevalence rates among community samples ranging from 12.5 to 23.6% [1, 2]. Particularly concerning is the documented association between NSSI, suicidality (i.e., suicidal ideation and attempt), and completed suicide, although the nature of these relationships is complex [3]. While NSSI and suicidal behaviors are phenomenologically distinct [4], a degree of overlap has been observed [5], and a history of NSSI remains one of the strongest predictors of later suicidal behavior [6, 7]. These observations support the conceptualization of NSSI along a continuum of self-harmful behavior in which suicide is the most severe endpoint [4]. However, between 59 and 72% of individuals who self-injure do not have suicidal thoughts at the time of self-injury [4]. Furthermore, despite high rates of co-occurrence between NSSI and suicide attempts among school-based adolescents [8, 9] a history of NSSI is absent among a proportion of individuals who ideate or attempt suicide [10]. Consequently, a fundamental question within the suicide prevention literature regards why some self-injurers engage in later suicidal behaviors, while others do not.

Other adolescent problem behaviors, including substance use, violence, and risky sexual activity have each been associated with suicidal behavior [11–13]. A significant corpus of research, much of it motivated by Problem Behavior Theory [14] describes the tendency for such risk behaviors to co-occur [15, 16]. Miller and Taylor [13] revealed that the number of problem behaviors increased the relative risk of suicidality. In comparison to adolescents with no problem behaviors, odds of a suicide attempt were 2.3 times greater among those with one problem behavior, 8.8 with two problem behaviors, and 18.3 with three problem behaviors, with odds increasing to 227.3 with six problem behaviors (i.e., disturbed eating, violence, binge drinking, illicit drug use, and risky sexual behavior). The 17% of youth with three or more problems accounted for 60% of all suicidal acts.

Recent research has considered whether the *nature* of co-occurrence (i.e., the type of behaviors exhibited by youth) represents an effective method of identifying those at risk for suicidality. Notably, behaviors across internalizing plus externalizing spectra appear to confer a higher risk of suicide than occurrence of either behaviour alone [17]. For example, adolescents with co-occurring depression and conduct disorder are at increased risk of suicide ideation and suicide attempts compared to adolescents reporting only depression or conduct problems [16]. Given NSSI (a manifestation of internalizing psychopathology) [18] is a risk factor for suicidality [3, 5] the notion that adolescents who self-injure and engage in other problem behaviors represent a subgroup with heightened risk for suicide holds intuitive appeal. Several studies have identified that NSSI co-occurs with substance use, disordered eating, and risky sexual behavior [19, 20] but few have examined whether co-engagement in problem behaviors, particularly externalizing behaviors, increases risk of suicidality among adolescent self-injurers.

Epidemiological studies suggest that firesetting occurs among 5–33% of adolescents recruited from community samples [21], and although research within adult psychiatric and forensic samples indicates firesetting often co-occurs with NSSI [22] only two studies to date have examined firesetting and NSSI concurrently among non-adjudicated adolescents. Martin et al. [23] found adolescent firesetters to be more likely than non-firesetters (including those engaging in other antisocial behaviors) to report histories of self-injury, and more likely to have attempted suicide. More recently, Tanner, Hasking, and Martin [24] observed that 52% of adolescent self-injurers had also engaged in firesetting, and almost half of these exhibited repeated firesetting (i.e., a lifetime history of three or more fires). In a follow-up study, adolescents reporting both NSSI and repeated firesetting were at significantly greater risk of suicidal behaviors than those engaging in either behavior alone [25]. Suicidal ideation was twice as likely among self-injurers who also set fires. Tanner et al. [25] posited a potentially synergistic effect between emotional and interpersonal distress (represented by NSSI) [10] and the capability for impulsive, aggressive behavior (represented by firesetting) [21].

However, not all young people with joint histories of NSSI and firesetting endorse suicidal thoughts or behaviors, suggesting the existence of underlying psychological factors that may differentiate youth who exhibit suicidality from those who do not. According to Joiner’s [26, 27] interpersonal theory of suicide (IPTS), suicidal behavior requires both the desire to die by suicide (involving perceptions of burdensomeness and thwarted belongingness) and the capacity to carry out lethal self-injury. NSSI may facilitate habituation to physical pain, emotional pain, and fear of dying, thereby increasing the capacity to consider or attempt suicide, as noted with risk-taking behavior [28]. Several plausible explanations exist for the increased likelihood of suicidality among adolescents with joint histories of NSSI and firesetting: Tanner et al. [25] identified that this group exhibited higher rates of psychological problems (known to increase suicidal desire) [18] and interpersonal stressors (reflecting interpersonal constructs of IPTS) [27], increased impulsivity and substance use (indicating impaired behavioral inhibition and decision making) [29] and more severe self-injury (representing habituation to NSSI) [27]. Similarly, poor coping strategies are often implicated in development of NSSI, suicidality, and general risk-taking behaviors [10], suggesting maladaptive coping may also underlie this relationship.

As noted by Tanner et al. [25], although the base rate of co-occurring NSSI and firesetting is low among school-based adolescents, this subgroup represents a significant minority (25%) of all adolescents with a past suicide attempt. Further understanding of these processes among a specific group identified as at elevated suicide risk may assist in identification of self-injurers at greatest risk for later suicidality [5], and explain increased rates of suicidality among adolescents engaging in both NSSI and firesetting. Our aim in this study was to explore, within this select group of adolescents, factors which differentiate those who report suicidal thoughts and behavior, from those who do not. For the present study, we hypothesized that a greater number of negative life events, mental health problems, impulsivity, poor coping, alcohol use, and more severe self-injury would be observed among adolescents reporting a prior suicide attempt, followed by those reporting ideation only, and then those reporting no suicidality. We also examined differences in reasons for living across levels of suicidality. While existing evidence suggests that different reasons for living have unique relationships with suicidality [30] this remains unexplored among youth engaging in multiple concerning behaviors. Reasons for living associated with suicidality may represent an ideal target for intervention.

## Method

### Participants

Participants were recruited from schools across five Australian state/territories in the final phase of a larger longitudinal study examining mental health among school-based adolescents. Forty-one of 115 schools contacted agreed to participate and explanatory statements/consent forms were distributed to parents of all students in selected grades (*n* = 14,841). Of these, 3,116 (21%) provided consent for their child’s participation, a rate consistent with previous Australian studies requiring active parental consent [31]. For a detailed description of the initial sample and sampling process, see Tanner et al. [24], and also data analysis below.

Of this initial sample, 77 participants engaged in both NSSI and high-frequency firesetting (52 females, 25 males) and comprised the sample for this study; participants were aged between 14 and 18 years (*M* = 16.04, *SD* = 0.86). Most were in their fourth (32.5%) or fifth (39%) year of secondary school. The majority (81.8%) were born in Australia and 1.3% identified as Aboriginal or Torres Strait Islander, consistent with the national profile for adolescents (86.3% Australian-born; 2.1% Indigenous) [32]. However both single-sex schools and consequently an over-representation of females were in the sample [32]. Consistent with the profile of the broader sample, participants were disproportionally recruited from areas of greater socio-economic advantage (*M* = 7.39, *SD* = 2.54, scale 1–10 whereby a low score indicates greater disadvantage) [33]. Most participants (78.90%) reported their parents were married. The majority (59%) had been in contact with mental health services and 39.53% self-reported a diagnosis of an emotional or behavioral problem, the most frequent being major depression (34.88%).

### Measures

#### Demographic information

In addition to age, gender, year at school, country of birth, and parental marital status (i.e., married, separated, divorced, etc.), participants provided their home post-code (i.e., zip code), used to estimate geographic remoteness (metropolitan, regional, rural and remote; ABS Remoteness Classification). Socio-economic status (SES) was computed from the ABS Index of Relative Socio-economic Advantage and Disadvantage (IRSAD) [33].

#### Suicidality

To assess suicidal ideation, participants were asked: “Have you ever thought about ending your life?” and indicated age at the most recent episode. For suicide attempt, participants were asked, “Did you ever try and end your life?” Respondents endorsing an attempt were asked to indicate method (i.e., “What did you do?”), and age at most recent incident.

#### Nonsuicidal self-injury

NSSI was assessed by Part A of the *Self-Harm Behavior Questionnaire* (SHBQ) [34], which measures intentional self-injury not identified as suicidal, and has been validated for use with adolescents [35]. Respondents were asked, “Have you ever hurt yourself on purpose?” and then requested to indicate the nature, frequency, and motivations for self-injury. Respondents rated severity of self-injury on a 4-point Likert scale (1 = not at all serious, 4 = life threatening). Respondents endorsing at least one lifetime episode were classified as having engaged in self-injury. Given that the use of single-item measure typically capture more general measures of self-harm (e.g., overdosing), participants were classified as engaging in NSSI only if direct methods were reported (e.g., cutting, burning, scratching, self-battery). To ensure assessment of nonsuicidal self-injury, participants were also excluded from analyses if reporting the same methods for NSSI and, in a subsequent section, suicide attempts.

#### Firesetting

Firesetting frequency was assessed with the question: “How many times have you set fire to something you weren’t supposed to?” Response options were 1–2 times, 3–5 times, 6 or more times, or never. In line with research suggesting 3 or more incidents of firesetting to be problematic [36], adolescents reporting 1–2 fires were excluded from final analyses. Participants were also asked: “How many times have you set fire to something resulting in damage?”

#### Mental health difficulties

Previous mental health difficulties were assessed by the Past Help-Seeking Experience component of the *General Help-Seeking Questionnaire* (GHSQ) [37]. Respondents were asked: “Has a doctor ever told you that you have an emotional or behavioral problem? If yes, what was the problem(s)?” Participants responding in the affirmative were classified as having a prior mental health problem.

#### Psychological distress

Psychological distress was measured with the *General Health Questionnaire* (GHQ-12) [38], a measure of current psychological functioning with an equal number of positively (e.g., “Been able to face up to your problems?”) and negatively phrased (e.g., “Felt that you couldn’t overcome your difficulties?”) questions. Respondents were asked to rate their functioning in the past few weeks on a 4-point Likert scale (1 = better than usual, 4 = much worse than usual*).* Cronbach’s alpha for scores in the present study was .89.

#### Personality characteristics

The BIS/BAS scale [39] is a 24-item measure assessing dispositional behavioral inhibition and behavioral activation. Responses are made on a 4-point Likert scale, summed to yield a global behavioral inhibition score and three separate behavioral activation scores: Drive, Fun Seeking and Reward Responsiveness. The behavioral inhibition subscale correlates with measures of susceptibility to punishment and harm avoidance (i.e., anxiety), while the behavioral activation subscales correlate with similar measures of extraversion, positive affectivity, reward seeking and impulsivity (Cronbach’s alphas for scale scores in the current sample were: Drive = 0.74; Fun Seeking = 0.68; Reward Responsiveness = 0.64; BIS = 0.67).

#### Negative life events

The *Adolescent Life Events Scale* (ALES) [40], is a measure of 20 potentially stressful life events relevant to adolescents. It asks whether each event happened in the past 12 months and/or more than 12 months ago. We used the ALES total score as well as lifetime rates of specific negative life events. Examples include, “Have you been bullied at school?” and “Have you been seriously physically abused?” The ALES has good reliability and validity [41]; Cronbach’s alpha for current the total score was 0.75.

#### Coping styles

Coping styles were assessed with the *Adolescent Coping Scale* (ACS) [42], which assesses 18 coping strategies rated on a 5-point scale (1 = *don’t do it*, 5 = *used a great deal*), summed to produce three coping styles: problem-solving, reference to others (i.e., approaching peers, professionals, etc.) and non-productive coping (i.e., avoidant behaviors). The ACS has been used extensively and has good validity and reliability [42]. Cronbach’s alphas for scores in our sample were 0.74 for “non-productive”, 0.76 for “problem-solving”, and 0.38 for “reference to others” coping.

#### Alcohol use

Alcohol use was assessed by the consumption subscale of the *Australian Alcohol Use Disorders Identification Test* (AusAUDIT) [43]. Three items assess quantity and frequency of alcohol consumption. AusAUDIT has good internal consistency and discriminant ability [43] and has been utilized across a range of community and clinical settings. Cronbach’s alpha for the current scale score was 0.89.

#### Reasons for living

Reasons for living were assessed using the *Brief Reasons for Living Inventory for Adolescents* (BRFL-A) [44], a 14-item measure of positive reasons for living with six adaptive categories: fear of Social Disapproval (FSD; concerns about what others would think of their actions), Moral Objections (MO; related to religious beliefs), Survival and Coping Beliefs (SCB; self-perceived coping ability), Responsibility to Family (RF; level of commitment to family), and Fear of Suicide (FS; fear of death and the act of suicide itself). Cronbach’s alphas for scores in our sample were 0.67 for FSD, 0.76 for MO, 0.76 for SCB, 0.80 for RF, 0.79 for FS.

### Procedure

Ethical clearance was obtained from affiliated universities and educational jurisdictions. Schools distributed explanatory statements and consent forms to parents/guardians outlining the purpose and procedures of the study. Children with parent/guardian consent completed the 1 h questionnaire at school. Participants were informed they could withdraw at any time, and supplied a unique code to facilitate confidentiality, yet enable identification in the event researchers identified imminent risk of harm. Adolescents who indicated current psychological distress, in the context of a negative outlook for the future and a suicide attempt within the last 12 months were identified to the school principal or school psychologist, who then implemented their schools’ procedures for assisting at-risk students. Researchers were present to clarify questions. On completion, participants received a pack with information about depression and mental health resources.

### Data analysis

Participants were excluded based on responses to the SHBQ and firesetting items: no history of NSSI or firesetting (*n* = 1501; 76.2% of initial sample), NSSI but no firesetting (*n* = 247; 12.5% of initial sample), and firesetting but no NSSI (*n* = 144; 7.3% of initial sample). Participants reporting both NSSI and repetitive firesetting (*n* = 77; 3.9% of initial sample) comprised our selected adolescent sample. These 77 participants were subsequently classified into three groups based on responses to questions regarding suicidal ideation and suicide attempt: adolescents with no suicidal ideation or attempt (*n* = 28; 36.4% of final sample), adolescents reporting ideation but no prior attempt (*n* = 34; 44.1% of final sample) and adolescents reporting a suicide attempt (*n* = 15; 19.5% of final sample). All adolescents reporting a suicide attempt also endorsed a history of suicidal ideation.

Following preliminary analyses, a multivariate analysis of variance (MANOVA) was conducted to assess differences in psychosocial functioning (i.e. reward sensitivity, psychological distress, coping, alcohol use, NSSI severity) across three levels of suicidality (1 = no suicidal ideation or attempt, 2 = suicidal ideation only, and 3 = suicide attempt).

Follow-up one-way analyses of variance (ANOVAs) were used to elucidate differences. Chi-square analyses were used to explore differences in specific negative life events across groups. A second MANOVA was performed to assess differences in the linear combination of the reasons for living scales across levels of suicidality. We chose to use separate MANOVAs for analyses due to modest cell sizes and the lack of significant correlation between sets of dependent variables (i.e., psychosocial characteristics and reasons for living). To address multiple analyses, Bonferroni corrections were applied to both MANOVAs and the analyses of individual life events, with resultant alpha levels of .003, and 0.002, respectively [45]. For all other analyses, an alpha level of .05 was utilized to indicate statistical significance.

## Results

### Statistical assumptions

In line with assumptions of performing MANOVA, linearity between pairs of variables across suicidality was assessed; inspection of the matrix of scatterplots indicated that the assumption of linearity was satisfied. Moderate correlations (0.11–0.32) between dependent variables suggested that multicollinearity would not interfere with interpretation of the results. Box’s M was significantly large, *p* = .25, satisfying the assumption of homogeneity of variance–covariance matrices. The assumption of equality of variance was met for all variables except for NSSI severity, Levene’s Test of Equality of Error Variances, *p* < .05; as a result, results regarding this variable are interpreted with caution.

### The nature and extent of problem behavior and suicidality

In our final sample, the age of onset for NSSI ranged from 8 to 17 years (*M* = 13.94, *SD* = 1.68). Half (52%) had self-injured in the last 6 months. Methods included cutting (45.91%), burning (10.81%), self-hitting or hitting head against hard objects (8.10%), and other (35%). Frequency ranged from 1 to 100 episodes, with 14.06% reporting one episode, 10.93% reporting two, 20.31% reporting three, and 54.68% reporting 4 or more episodes. For firesetting behavior, more than half (53.23%) had set a fire 6 or more times; the remaining 46.77% had set 3–5 fires. Most (61%) had not set a fire resulting in damage, 26.34% had done so on 1–2 occasions, 6.61% on 3–5 occasions, and 5.85% on 6 or more occasions. Of those with suicidal ideation (63.21%), more than half (54.50%) had last thought of ending their life in the previous 12 months. Similarly, of those reporting a past suicide attempt (19.50%), more than half (53.33%) had attempted in the last 12 months. Methods of suicide attempt included cutting arms and wrists (40%), drug overdose (33.33%), hanging (13.33%), and other (19.33%). Table 1 provides descriptive information on demographic and psychosocial characteristics of each group.

**Table 1:** Descriptive statistics for variables of interest across suicidality level

	No Suicidality *n* = 28	Ideation only *n* = 34	Attempt *n* = 15
Demographics			
Gender			
Male	12 (42.9%)	9 (26.5%)	4 (26.7%)
Female	16 (57.1%)	25 (73.5%)	11 (73.3%)
Age (SD)	16.07 (0.98)	16.15 (0.74)	15.73 (0.88)
SES^a^	7.69 (2.83)	7.18 (2.40)	7.25 (2.31)
Negative life events			
Total ALES score	34.01 (5.63)	37.73 (5.67)	40.47 (5.22)
Difficulty keeping up with school work	27 (96.42%)	32 (94.12%)	15 (100%)
Difficulty making or keeping friends	19 (67.86%)	21 (63.64%)	11 (73.33%)
Serious arguments/fights with friends	23 (82.14%)	30 (88.23%)	11 (73.33%)
Serious problems with boy/girlfriend	13 (46.43%)	18 (52.94%)	9 (60%)
Bullying victimization	17 (60.71%)	24 (70.59%)	14 (93.33%)
Parental separation or divorce	6 (21.43%)	8 (23.53%)	9 (60%)
Serious conflict with parents	21 (75%)	30 (88.23%)	13 (86.67%)
Serious conflict between parents	11 (42.31%)	23 (71.87%)	11 (73.33%)
Serious illness/accident self or family	22 (78.57%)	26 (76.47%)	12 (80%)
Serious illness/accident close friends	10 (35.71%)	19 (57.57%)	7 (46.67%)
Serious physical abuse	1 (3.57%)	7 (20.59%)	8 (53.33%)
Trouble with the police	7 (25%)	9 (26.47%)	5 (33.33%)
Death among immediate family	2 (7.14%)	4 (11.76%)	4 (26.67%)
Death of someone close	16 (57.14%)	28 (82.35%)	11 (73.33%)
Family or friend completed suicide	3 (10.71%)	9 (26.47%)	14 (93.33%)
Family self-harm or suicide attempt	10 (35.71%)	8 (23.53%)	5 (33.33%)
Friend self-harm or suicide attempt	20 (71.43%)	25 (73.53%)	14 (93.33%)
Worries about sexual orientation	9 (32.14%)	10 (29.41%)	8 (53.33%)
Sexual assault	1 (3.57%)	11 (32.35%)	4 (26.67%)
Other distressing event	12 (42.86%)	19 (55.88%)	13 (86.67%)
Psychological characteristics			
BAS Drive	11.44 (2.16)	11.16 (2.60)	9.31 (2.81)
BAS Reward	16.76 (1.39)	16.64 (2.79)	14.85 (4.52)
BAS Fun	13.36 (2.38)	13.77 (2.10)	12.133 (3.16)
BIS	18.72 (5.41)	22.39 (4.25)	23.15 (3.74)
Psychological distress	24.96 (7.10)	31.22 (8.99)	33.40 (8.39)
Problem solving coping	63.36 (12.16)	55.06 (16.02)	46.61 (14.73)
Reference to others coping	42.80 (12.83)	44.84 (14.11)	35.00 (10.41)
Non-productive coping	58.22 (10.57)	62.81 (12.13)	66.32 (7.23)
Alcohol use	7.36 (4.02)	7.11 (3.40)	7.07 (4.15)
NSSI severity	1.36 (0.54)	1.65 (0.54)	2.50 (0.83)
Reasons for living			
Fear of Social Disapproval	10.41 (3.51)	11.09 (4.37)	11.60 (5.19)
Moral Objections	8.25 (4.48)	6.41 (3.88)	5.40 (3.54)
Survival and Coping Beliefs	13.96 (3.57)	11.79 (3.14)	7.93 (3.83)
Responsibility to Family	14.43 (3.90)	13.53 (3.86)	11.13 (5.40)
Fear of Suicide	5.68 (3.50)	6.76 (4.09)	4.93 (2.79)

^a^Refers to ABS Index of Relative Socio-economic Advantage and Disadvantage (IRSAD; ABS, 2006, 2008).

### Preliminary analyses

Gender (*p* = .35), age (*p* = .30), and SES (*p* = .76) were not related to level of suicidality and therefore were not controlled for in subsequent analyses. Prior diagnosis of a mental health problem was significantly related to suicidality (*p* < .05). We included a continuous measure of psychological distress (i.e., the GHQ; *p* < .001) in the multivariate model—partly due to restrictions regarding use of dichotomous variables in MANOVA analyses—but also to obtain meaningful representation of severity of mental health problems among participants.

### Differences in psychosocial functioning across levels of suicidality

A one-way MANOVA revealed suicidality was related to psychosocial characteristics, Wilks’ λ = .55, *F*(12, 138) = 3.98, *p* < .001. Specifically, behavioral inhibition, negative life events, psychological distress, and NSSI severity were lowest among adolescents with no suicidality and increased with levels of suicidality (Table 2). Conversely, BAS Drive and problem-solving coping were highest among adolescents with no suicidality and decreased with levels of suicidality. Adolescents reporting suicidal ideation endorsed more anxiety, more negative life events, and higher psychological distress than those reporting no suicidality (all *p* < .05); scores were also greater among adolescents with a suicide attempt relative to those reporting no suicidality (all *p* < .01). Adolescents with no suicidality reported higher BAS Drive than those endorsing suicidal ideation or attempt (both *p* < .05); these adolescents also reported greater use of problem-solving coping than those with a suicide attempt (*p* < .01), but not suicidal ideation. No differences in BIS, negative life events, psychological distress, BAS Drive, or problem-solving coping were observed between adolescents with ideation and those with a prior suicide attempt (all *p* > .05). However, adolescents reporting a prior suicide attempt engaged in more severe NSSI than those reporting suicidal ideation and those reporting no suicidality (both *p* < .001).

**Table 2:** Univariate Analysis of Variance for Significant Variables across Level of Suicidality

	Sum of squares	df	Mean square	*F*	Partial Eta Squared
Psychological characteristics					
BIS	247.07	2	123.54	5.77**	.15
BAS Drive	42.35	2	21.18	3.42*	.09
Negative life events	415.64	2	207.82	7.20**	.19
Psychological distress	902.20	2	451.10	6.67**	.16
Problem-solving coping	2,510.51	2	1,255.25	5.98**	.15
NSSI severity	9.84	2	4.92	16.35***	.27
Reasons for living					
Survival and coping beliefs	335.94	2	167.97	14.27***	.28

* *p* < .05; ** *p* < .01; *** *p* < .001.

### Specific negative life events and suicidality

In addition to total negative life events, we examined whether *specific* negative life events were reported across increasing levels of suicidality (Table 3). A series of Chi-square analyses revealed that adolescents reporting suicidal ideation, relative to those with no history of suicidality, were more likely to have experienced serious physical abuse or sexual assault. Adolescents reporting a suicide attempt were more likely than those with no history of suicidality to report bullying victimization, serious physical or sexual abuse, and the suicide of a friend or family member. These adolescents were also more likely to have experienced serious physical abuse or the suicide of a friend or family member, than adolescents reporting suicidal ideation only.

**Table 3:** Differences in prevalence of specific negative life events across level of suicidality

	Ideation only^a^		Attempt^a^		Attempt^b^	
	χ^2^	OR (95% CI)	χ^2^	OR (95% CI)	χ^2^	OR (95% CI)
Negative life event^c^						
Bullying victimization	0.66	0.64 (0.22–1.85)	5.16*	9.06 (1.04–79.36)	3.09	5.83 (0.67–50.53)
Serious physical abuse	3.96*	5.00 (1.24–31.20)	14.61***	20.78 (3.06–140.92)	5.25*	4.41 (1.19–16.36)
Family or friend suicide	2.44	2.71 (1.41–10.39)	14.50***	16.67 (3.34–83.24)	7.08**	5.55 (1.49–20.72)
Sexual assault	8.15**	8.97 (1.50–53.65)	5.07*	7.17 (1.01–51.54)	0.15	1.25 (0.34–4.58)

* *p* < .05; ** *p* < .01; *** *p* < .001.

^a^Reference group = No history of suicidality.

^b^Reference group = Ideation only.

^c^Only significant differences displayed.

### Differences in reasons for living across levels of suicidality

Adolescents with varying levels of suicidality differed on a linear composite of scores on RFL subscales, Wilks’ λ = .62, *F*(10, 136) = 3.61, *p* < .001. Specifically, groups differed on their scores for survival and coping beliefs (Table 3); adolescents who had attempted suicide reported lower survival and coping beliefs than those who ideated (*p* < .001) and those with no suicidality (*p* < .05). No difference in survival and coping beliefs was observed between adolescents who ideated and those with no suicidality (*p* > .05).

## Discussion

Co-occurrence of internalizing and externalizing behaviours appears to increase risk of suicidal ideation and behaviour [16, 17]. Consistent with this, we have previously noted that adolescents who engage in both NSSI and firesetting are at elevated risk [24, 25]. The current study builds on this previous work to examine factors that potentially confer this risk. We aimed to identify which psychosocial characteristics of a sub-group exhibiting both NSSI and firesetting differed across levels of suicidality. We expected indicators of psychosocial dysfunction to be greater across increasing levels of suicidality. Overall, support for this hypothesis was observed.

### Suicidality and mental health difficulties

The rate of suicidal ideation (63%) and attempt (19%) among the current sample is higher than in incarcerated youth (e.g., 19.2% ideation, 8.4% attempt [46], and comparable to reports within adolescent psychiatric samples (e.g., 58% ideation, 29% attempt) [47]. Our findings extend our previous work by suggesting that, in addition to a previous diagnosis of a mental health problem [24], adolescents engaging in both NSSI and firesetting and exhibiting suicidal tendencies are experiencing ongoing and *current* psychological distress. Collectively, these observations add to existing research on outcomes for multi-problem youth [13, 16] by demonstrating that co-occurring NSSI and firesetting is associated with significant psychological impairment and suicidality during adolescence. Findings highlight the importance of addressing mental health problems in suicide prevention efforts among this subgroup.

### Negative life events and suicidality

Consistent with past research highlighting the role of life stressors in development of suicidal thoughts and behaviors [48], negative life events emerged as an indicator of suicidal ideation and attempt. Notably, interpersonal or violent victimization (i.e., experiences of serious physical or sexual assault) increased the likelihood of suicidal ideation among our sample. In addition to experiences of physical or sexual assault, adolescents attempting suicide were more likely to have been bullied, or lost a friend or family member to suicide, than those reporting suicidal thoughts in the absence of an attempt. Indeed, experiences of abuse, an inability to effectively handle interpersonal stressors such as bullying, and exposure to suicidal behavior have each been identified as “tipping points” for suicidal behavior [49]. However, our findings extend this knowledge by suggesting that while experiences that threaten physical integrity or challenge one’s sense of safety and security (i.e., physical and sexual abuse) lead adolescents to consider ending their life, ongoing interpersonal difficulties (i.e., bullying victimization) and exposure to completed suicide in peer networks and family are factors that may prompt these individuals into action.

### Psychological characteristics and suicidality

Personality characteristics related to anxiety (i.e., Behavioral Inhibition) and persistence in the pursuit of goals (i.e., drive) were differentially related to suicidality. These findings accord with prior research indicating adolescents who are sensitive to negative experiences, or those who perceive themselves as unable to pursue goals, to be at higher risk of suicidal behavior [50, 51]. Despite trends in the literature associating impulsivity-related personality traits and alcohol use with adolescent suicide [52, 53], we found no differences in these variables across levels of suicidality. This may be attributable to the measures of impulsivity and alcohol use employed in the current study. Although the Fun-Seeking subscale of the BIS/BAS is highly correlated with well-validated measures of impulsivity [39], other evidence suggests the scale to have greater specificity for measuring trait-like tendencies to pursue novelty and reward (e.g., sensation-seeking) [54]. Tanner et al. [24] observed Fun-Seeking to predict co-occurring NSSI and firesetting, suggesting the sensation- seeking aspect of impulsivity may relate to engagement in problem behaviors, while other impulsivity-related traits (e.g., negative urgency, the tendency to act rashly when experiencing negative affect) [55] may be more salient in predicting suicidality among high-risk youth. Similarly, the AusAUDIT assesses the frequency and quantity of alcohol consumption rather than problematic use, thus, it is possible that while underage alcohol consumption relates to engagement in multiple problem behaviors, it is the problematic use of alcohol that elevates the risk of suicidal ideation and attempt among multi-problem youth [56]. Additional research is required to examine these hypotheses in greater detail.

The use of maladaptive coping strategies has long been implicated in suicidal thoughts and behaviors [57], thus, it is interesting that in the current study a lack of problem solving, rather than the use of non-productive coping strategies (i.e., avoidance or disengagement), was related to suicidality. However, this finding is consistent with research identifying that an inability to generate solutions in the context of life stressors or psychological distress is a key deficit among individuals who have considered or attempted suicide [58]. It must also be noted that although differences in non-productive and reference to others (i.e., use of external supports) coping did not reach statistical significance in the present study, inspection of mean scores indicated a greater use of non-productive strategies, and a lower use of reference to others coping, as level of suicidality increased. It is possible that significant differences would emerge in replication studies with larger samples. However, our current findings regarding coping beliefs (discussed below) may provide an alternative explanation for these findings.

Adolescents with a past suicide attempt reported more severe self-injury (i.e., greater resultant harm, such as requiring medical attention) than those reporting suicidal ideation only, an observation lending support to the habituation hypothesis (i.e., that repeated NSSI desensitizes….; Joiner [27]). Interestingly, frequency of NSSI did not differ significantly between adolescents reporting suicidal ideation and those who had attempted suicide (*M*_episodes_ = 5.8 vs. 4.8, respectively). Several researchers have found frequency of self-injury to predict suicide attempts [20], others failing to observe similar relationships [10]. Given the current sample comprised youth with NSSI and firesetting, it is possible that frequency of NSSI is only predictive of suicide attempts so far as it increases acquired capability for suicide, but once this capability is established (i.e., in subgroups of adolescents engaging in multiple problem behaviors), the salience of NSSI frequency in predicting suicide diminishes. It is also possible that the more medically severe self-injury reported by adolescents with a prior suicide attempt in the current study represents ‘trialing’ of suicidal behavior (i.e., an episode of self-injury with ambiguous intent) when existing attempts to manage distressing experiences (e.g., engagement in multiple problem behaviors) are no longer effective. Research examining the role of frequency versus severity in the NSSI/suicidality nexus, which also clarifies self-injurious intent, may assist in addressing these hypotheses.

### Reasons for living as protective factors against suicidality

Finally, the current study examined potential protective factors against suicidality (i.e., reasons for living) among adolescents exhibiting co-occurring NSSI and firesetting. Although it is essential to consider both risk and protective factors in order to accurately evaluate suicide risk [59], the majority of research efforts to date have focused on identifying risk factors for suicidality; thus, our finding that survival and coping beliefs may buffer the risk of suicide attempt among at-risk adolescents represents an important addition to existing suicide prevention literature. Notably, when considered alongside our results regarding coping style, which implicated a lack of problem-solving skills rather than use of avoidant coping strategies, the current findings suggest that an adolescent’s perception of their ability to cope with or generate solutions to problems (i.e., self-efficacy related to coping) may play a more salient role in protecting against suicidality. Consistent with this hypothesis, recent development of the Self-Efficacy to Avoid Suicidal Action scale (SEASA) [60], is an important step in predicting suicide attempts. Considered in this context, our current findings suggest the study of coping-related self-efficacy may add to understanding of modifiable factors to inform interventions with suicidal adolescents.

### Implications

Although tentative given the small sample and the limitations noted below, a number of our findings support the habituation hypothesis [27] in explaining links between NSSI and suicidality, namely, (a) the high rate of suicidality among a subgroup of adolescents engaging in problem behaviors likely to involve pain and/or fear, (b) the implication of negative life events involving physical pain and fear (i.e., physical abuse, sexual assault, and bullying victimization) and (c) the increase in medical severity of NSSI observed among adolescents with a suicide attempt.

Taken together, with results of our previous work [24, 25], results of our study provide valuable insights into clinical suicide risk assessment among subgroups of adolescent self- injurers. Specifically, our findings indicate a number of commonly cited suicide risk factors—impulsivity, substance use, and maladaptive coping—may not be reliable predictors of suicidal thoughts or attempts among most at-risk adolescents. Similarly, while research examining NSSI characteristics and acquired capability for suicide has focused on frequency of self- injury [3], current findings indicate that NSSI severity may be better at identifying which adolescents are most likely to act on thoughts of suicide. We recommend mental health professionals enquire about seriousness of wounds following self-injury when assessing risk for suicide.

Our findings indicate that while experiences of victimization and psychological distress are observed among adolescents with suicidal ideation, ongoing interpersonal difficulties, threats to physical integrity (i.e., more severe NSSI and physical abuse), and exposure to suicide in close relationships may help differentiate adolescents more likely to act on thoughts of suicide. These factors may assist clinicians in identifying adolescents requiring a thorough risk assessment and suicide prevention plan.

Finally, this study supports the importance of incorporating resilience factors into suicide risk assessment, as well as early intervention and prevention efforts. Developing coping and problem-solving ability, and possibly more importantly, addressing beliefs regarding an individual’s ability to cope with distressing experiences (e.g., cognitive restructuring that directly targets suicidal ideation) [61] appear to be promising interventions to reduce suicide attempts among at-risk adolescents. While it must be noted that the aforementioned findings relate specifically to youth engaging in NSSI and firesetting, future research is encouraged to examine whether similar relationships exist among adolescents engaging in NSSI and other externalizing behaviors (e.g., violence, substance use, etc.).

### Limitations

The cross-sectional nature of this study precludes conclusions regarding causality. It is possible that suicidal ideation or an attempt preceded engagement in NSSI and/or firesetting; these behaviors may represent alternate expressions of psychological distress or attempts to distract from suicidal tendencies [10]. Although most research suggests NSSI precedes suicidal behavior [3, 5], temporal analyses could elucidate the direction of relationships between problem behaviors, psychosocial variables, and suicidality. Researchers are encouraged to conduct ongoing longitudinal examination of the aforementioned findings in order to further our understanding of the relationship between co-occurring problem behaviors and suicidality.

The number of participants reporting suicidal ideation (n = 34) or attempt was too small (*n* = 15) to reliably conduct more complex analyses, and explore more intricate associations between factors of interest and suicidality. As noted earlier, we chose to focus on a small, select, group of young people we previously observed to be at heightened risk of suicidal thoughts and behavior, with a view to differentiating those who report suicidal thoughts and behavior from those who do not. However, the inclusion of a large number of variables within such a small sample reduces power and necessitates the use of caution in interpreting the current findings. In future, researchers may benefit from oversampling within this population in order to obtain the required power to conduct more complex statistical analyses. Further, it would be interesting to explore reasons for living in a larger sample of youth reporting suicidal ideation, but no attempt, to ascertain which might be protective factors among young people contemplating suicide.

Related to this, in order to increase our sample size we included adolescents who had only engaged in NSSI once within our sample. Previous work suggests young people who engage in NSSI at least 4 times are most likely to report adverse outcomes [62], consistent with proposed DSM criteria for NSSI (NSSI on at least 5 days in the last year) [63]. Yet, while adolescents exhibiting fewer than four episodes of NSSI might be considered to engage in relatively mild NSSI, our data suggest that if they also engage in repetitive fire-setting their risk of suicidal behavior is elevated. Consequently, we cautiously suggest that assessment of behavioral issues, such as firesetting, be conducted even when only mild forms of NSSI are exhibited. Still, restricting the definition of NSSI, yet oversampling to recruit a larger total sample, and particularly a larger sample reporting suicidal thoughts and behaviors, would enable the inclusion of additional analyses, such as the relevance of the frequency and severity of both NSSI and firesetting to suicidality.

An additional limitation of the present study involved reliance on self-report assessments to measure engagement in problem behaviors and suicidality. In particular, previous studies have highlighted challenges in regards to the validity of self-reported suicidal behavior [64]. Future research utilizing multi-informant methods might offer additional utility in examining the relationship between problem behaviors and suicidality among youth. In addition, inclusion of a more detailed measure of suicidal ideation (e.g., the Scale for Suicidal Ideation) [65] would enable a more nuanced examination of the presence of suicidal ideation among this subgroup, such as the distinction between passive desire and specific plans for suicide.

Although a small study with noted limitations, the present study is the first to examine suicidality among a subgroup of school-based adolescents engaging in both NSSI and firesetting. While not a high-prevalence group of young people, clinicians can be mindful of elevated suicide risk among this select sub-group, and factors which might exacerbate or mitigate this risk. Findings suggest that exposure to experiences involving pain and fear (e.g., problem behaviors, NSSI of increasing severity, and personal victimization) might underlie the relationship between NSSI and suicidality in adolescence, but further work is required to test this proposition. The role of mental health problems and self-perceived ability to cope may also be implicated in the development of suicidality among multi-problem youth. Further exploration of the nuances of these relationships would be assisted by subsequent research with larger samples, with the goal of identifying and developing suicide prevention initiatives among select subsets of self-injurers.
